# Cholesterol Emboli Syndrome Following Angioplasty: A Case Report and Literature Review

**DOI:** 10.7759/cureus.90301

**Published:** 2025-08-17

**Authors:** Reeju Maharjan, Ozo Akah, Ankur Gangahar, Ewuradjoa Ayirebi-Acquah, Anshi Jain, Arlette Villalobos

**Affiliations:** 1 Neurology, V. N. Karazin Kharkiv National University, Kharkiv, UKR; 2 Neurology, California Institute of Behavioral Neurosciences and Psychology, Fairfield, USA; 3 Internal Medicine, Carnegie Mellon University (CMU), Houston, USA; 4 Medicine, Government Medical College, Amritsar, Amritsar, IND; 5 Internal Medicine, Lekma Hospital, Accra, GHA; 6 Community Medicine, K. J. Somaiya Medical College and Research Center, Mumbai, IND; 7 Surgery, Ponce Health Sciences University, Ponce, USA

**Keywords:** angioplasty, atherosclerotic plaques, blue toe syndrome, cholesterol emboli syndrome (ces), embolization, ischemia, skin biopsy, vasculitis

## Abstract

Cholesterol emboli syndrome (CES) occurs when cholesterol crystals from atherosclerotic plaques embolize to distal arteries, often after invasive procedures like angioplasty. We report a 67-year-old man with hypertension, diabetes, and hyperlipidemia, who developed painful discoloration of his left toes following angioplasty for the management of myocardial infarction. Diagnosis of CES was confirmed by clinical findings, imaging, and a skin biopsy showing leukocytoclastic vasculitis. The patient was treated with anticoagulation, analgesics, and a vascular surgery consultation. This case underscores the importance of early recognition and management of CES to prevent further complications.

## Introduction

Cholesterol emboli syndrome (CES), also known as cholesterol crystal embolization (CCE), atheromatous embolization, or atheroembolism, is a condition in which cholesterol crystals (CCs) dislodged from atherosclerotic plaques - along with fibrin and platelets - travel from larger arteries to smaller arteries, leading to ischemia [1]. This syndrome is commonly observed after intra-arterial interventions such as percutaneous coronary intervention (PCI) or angioplasty [2].

CES comprises various symptoms, such as livedo reticularis, leg and/or foot pain, cyanosis (blue toe syndrome), kidney impairment, eosinophilia, present foot pulses, ulceration, purpura, and painful red nodules [3]. Blue toe syndrome refers to the blue or violet discoloration of the toe tip, and it is characterized by a cool, painful, cyanotic toe with a well-perfused foot [4]. As a result of an inflammatory response, partially occluded arterioles can eventually become completely occluded [2]. Approximately 80% of CES cases are iatrogenic, and spontaneous onset is rare (1.9%-13%) [5]. Generally, the main cause is a complication following an invasive arterial procedure. We present a case of a 67-year-old man with a history of hyperlipidemia, diabetes, and hypertension, who developed painful discoloration of the left toes after angioplasty for myocardial infarction. The diagnosis of CES was confirmed, and treatment included anticoagulation, analgesics, and a vascular surgery consultation.

## Case presentation

A 67-year-old male presented to the Emergency Department with a three-day history of progressive, painful discoloration of the toes on his left foot. The symptoms began as bluish discoloration of the second toe and extended to involve adjacent digits, associated with constant burning pain exacerbated by movement or limb dependency. He denied trauma, cold exposure, or recent infections, and reported no fever, chest pain, or neurological symptoms. One week earlier, he had undergone an uncomplicated PCI with balloon angioplasty for stable coronary artery disease and was discharged on dual antiplatelet therapy, a high-intensity statin, and his usual antihypertensive and antidiabetic medications. His past medical history included hypertension, type 2 diabetes mellitus, and hyperlipidemia. He was a retired schoolteacher, a former smoker with a 20 pack-year history, and lived independently with his spouse.

**Figure 1:**
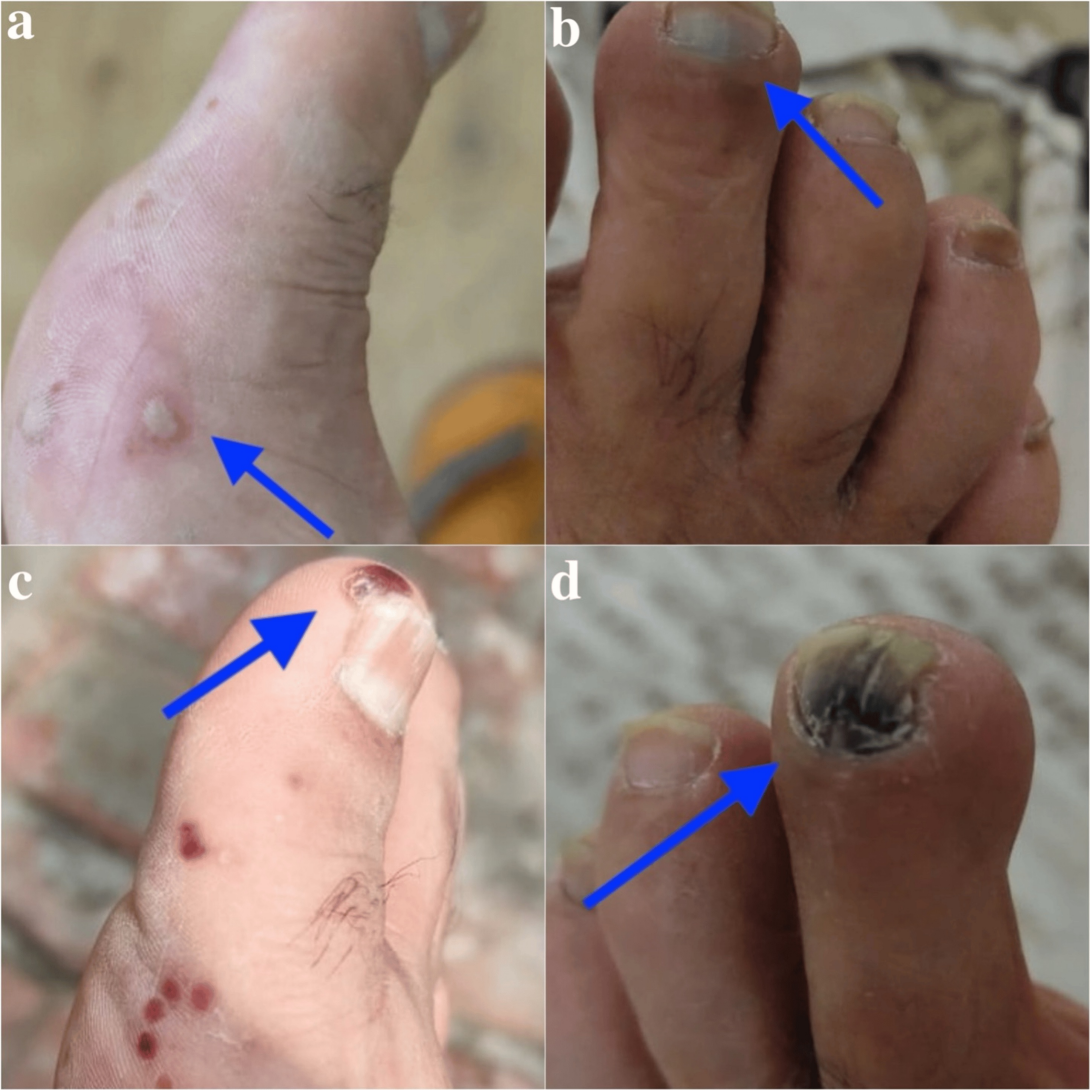
Clinical progression of cholesterol embolization syndrome (a, b) Initial Presentation - Livedo reticularis on the dorsal foot, purpuric rash on the lower legs, and intermittent discoloration like blue toe syndrome, raising suspicion for underlying vasculitis. (c, d) After 3 Months - Worsened discoloration, increased pain, and an expanded purpuric rash on the lower legs.

On examination, he was afebrile, alert, and hemodynamically stable. Cardiovascular examination revealed regular heart sounds with no murmurs, strong femoral pulses bilaterally, and diminished dorsalis pedis and posterior tibial pulses on the left compared to the right. The left foot demonstrated marked cyanosis of the second toe, with a mottled, shiny skin surface and early necrosis at the tips of the second and third toes. Livedo reticularis was present over the lateral aspect of the foot, and capillary refill time in the affected toes exceeded four seconds. The skin distal to the midfoot was cool to the touch, with mild non-pitting edema, reduced sensation to light touch, and preserved motor function. A purpuric rash was noted on the anterior aspects of both lower legs (Figures 1a-1b). No calf tenderness, swelling, or ulcers were present, and examination of other systems was unremarkable.

At the three-month follow-up, the patient returned with worsening symptoms. The discoloration of the left toes had progressed, with increased cyanosis and more pronounced necrosis at the tips. Pain intensity had increased to 9/10. The purpuric rash had expanded, and larger areas of necrosis were noted on the lower legs. Livedo reticularis on the lateral aspect of the left foot had become more pronounced, suggesting worsening ischemic changes. These findings raised concerns for progressive CES, a complication following angioplasty, necessitating continuous monitoring and further diagnostic assessment (Figures 1c-1d).

**Figure 2:**
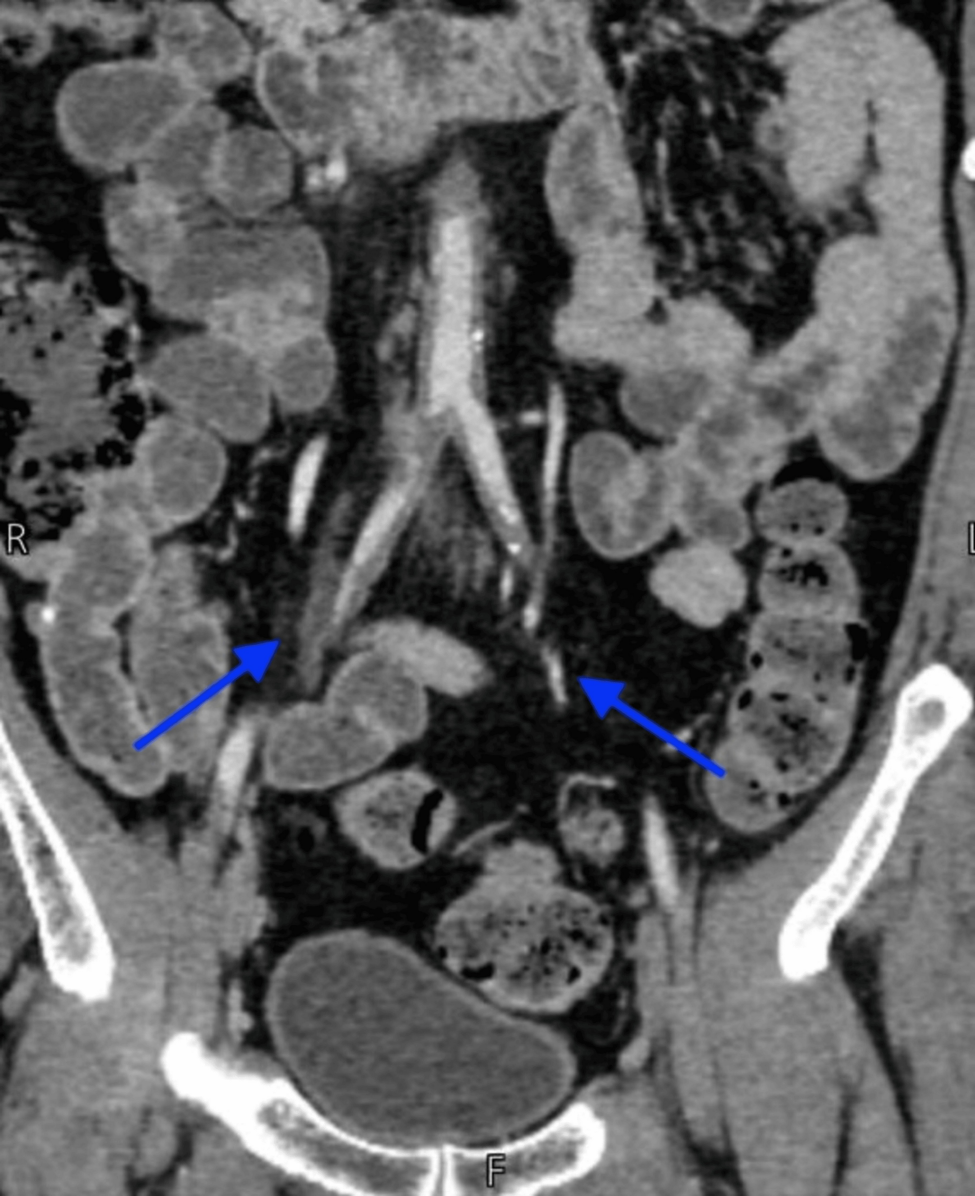
CT angiography with digitally marked arrows highlighting proximal occlusion of aortic branch vessels

To assess vascular status and potential embolic phenomena, Doppler ultrasound and CT angiography were conducted (Figure 2). Doppler ultrasound of the lower extremities revealed markedly reduced digital arterial flow, with significant proximal arterial blockages, suggesting an embolic rather than a purely local thrombotic process. CT angiography demonstrated proximal occlusion of aortic branch vessels - a finding consistent with CCE originating from a proximal source rather than isolated peripheral thrombosis. MRI of the left foot demonstrated soft tissue edema in the digits, without evidence of acute muscle infarction, indicating ischemia confined to the microvasculature - an expected pattern in CES, where small- and medium-sized vessels are occluded by CCs, unlike the larger-vessel thromboembolism seen in classic blue toe syndrome.

A skin biopsy of the purpuric rash revealed leukocytoclastic vasculitis, characterized by fibrin deposition and inflammatory cell infiltration within the dermal vessels. However, no cholesterol clefts were identified, making the histopathological confirmation of CES inconclusive. Additional laboratory investigations showed elevated inflammatory markers, including a C-reactive protein (CRP) level of 38.7 mg/L (normal: <5 mg/L), and an erythrocyte sedimentation rate (ESR) of 54 mm/hr (normal: <20 mm/hr). Given the systemic nature of CES, renal function markers were monitored, including serum creatinine and estimated glomerular filtration rate (eGFR); however, no significant deterioration was observed. Further autoimmune workup, including anti-neutrophil cytoplasmic antibody (ANCA) testing, was negative, effectively ruling out ANCA-associated vasculitis.

The primary differential diagnosis included blue toe syndrome vs. CES. Blue toe syndrome typically results from small arterial occlusions due to thromboembolism, rather than cholesterol embolization, and often lacks systemic involvement. The presence of livedo reticularis, purpuric rash, and progressive ischemic changes suggested CES over blue toe syndrome. However, the absence of cholesterol clefts on histology remained a diagnostic limitation.

The patient was admitted for close monitoring and symptom management. He was started on analgesics for pain control, but anticoagulation therapy was not initiated due to its controversial role in CES. Anticoagulation was avoided, as it may worsen CES by destabilizing plaques, potentially increasing distal embolization. Instead, statin therapy was optimized to promote plaque stabilization. A vascular surgery consultation was obtained, and conservative management with risk factor modification was emphasized. Given the progressive necrosis, discussions regarding potential surgical intervention, including toe amputation, were initiated.

## Discussion

CCE is a rare but serious complication of PCI, although it is mainly iatrogenic [6]. Positive predictors of CCE were age ≥70 years (68% vs. 59%, p = 0.012), aortic aneurysm (23% vs. 7.2%, p < 0.001), and a femoral approach (71% vs. 45%, p < 0.001), whereas a negative predictor of CCE was the use of an inner sheath (63% vs. 77%, p < 0.001) [6]. The incidence of CCE in patients who underwent intravascular procedures, including PCI, is reported to be 0.6% to 0.9% [6]. This case illustrates a classic presentation of post-angioplasty CES, manifesting as blue toe syndrome and skin findings consistent with vasculitis. In general, the classic triad of renal failure, peripheral CCE, and a precipitating event suggests the diagnosis of CCE. This can be confirmed by biopsy of the target organs [7]. The patient's recent angioplasty likely led to the dislodgment of CCs from atherosclerotic plaques, resulting in embolization to the distal vasculature. The combination of cyanosis, ischemic pain, and skin changes underscores the need for timely recognition and management of this complication.

The main risk factors for developing CES include advanced age, male sex, coronary artery disease, diabetes mellitus, hypertension, and a history of smoking. This condition can progress to end-stage renal failure and ultimately result in death if left untreated. Waitupu et al.'s study examined the complex relationship between CCE, the immune system reaction, and atherosclerosis [8]. CCE results from ruptured atherosclerotic plaques, triggering an immune response. During this immune response, activation of the NLRP3 inflammasome occurs, which further leads to the secretion of proinflammatory cytokines, exacerbating vascular inflammation and atherogenesis [8].

When macrophages phagocytose CCs, these crystals can disrupt the lysosomal membrane, leading to the release of cathepsin B into the cytoplasm [8]. The activated NLRP3 inflammasome then leads to the activation of caspase-1, which processes the proinflammatory cytokines interleukin-1 beta (IL-1β) and interleukin-18 (IL-18) into their mature forms [8]. Once activated, IL-1β and IL-18 play pivotal roles in promoting inflammation. IL-1β is a potent mediator of fever and inflammation, and has been directly linked to the development of atherosclerotic disease [8]. Furthermore, the activated NLRP3 inflammasome promotes the recruitment of additional immune cells to the site of inflammation, amplifying the inflammatory response. This process plays a crucial role in atherogenesis, contributing to the worsening of vascular disease and the progression of CES. These findings underscore the central role of NLRP3 inflammasome activation in the inflammatory pathway associated with CCE and atherosclerosis, emphasizing the need for targeted therapeutic strategies to manage this condition effectively.

In the study by Tokatli et al., painful, blue-colored macular lesions were observed on both hands and feet, similar to our findings. Purple or blue discoloration of the foot, known as blue toe syndrome, is synonymous with CES [9]. Similarly, in the study by Otsubo et al., bilateral acrocyanosis and diffuse necrotic lesions, covered with black scabs, appeared on the patient’s toes 10 days after undergoing percutaneous transluminal coronary angioplasty (PTCA). The patient complained of pain in the right third, fourth, and fifth toes, as well as the left fourth and fifth toes. Biopsy specimens from the skin of his right third toe demonstrated capillary growth, dilatation, and fibrosis of the skin corium. Needle-shaped CCs were found in the intraluminal clefts of small capillaries, characteristic of cholesterol embolisms [10]. Similarly, in the study by Nhiri and Bazid, an 84-year-old man developed new-onset cyanosis in his feet, affecting both the toes and distal fingers, following coronary angiography and stent implantation 14 days prior [11].

Further supporting these findings, Hendrickx et al. observed a patient who developed severe calf pain five days after undergoing PTCA, diagnosed as deep thrombophlebitis and treated with subcutaneous injections of Calciparin. In the following days, the patient’s condition worsened, and the appearance of livedo reticularis, acrocyanosis of the feet, and large, tender ecchymotic nodules appeared, particularly in the lower abdomen and limbs, where they exhibited a reticular vascular pattern. Over time, these lesions underwent central necrosis with the formation of black eschars [12]. Similarly, Akpala et al. reported a patient who, approximately 10 weeks after coronary bypass surgery, began experiencing painful blue discoloration of the toes. This gradually worsened, and a CT angiogram revealed diffuse atherosclerotic disease with multiple areas of stenosis in the lower extremities. The patient was started on oral rivaroxaban, and, on examination, there were purple discolored patches and papules with ulceration involving several toes [13]. To investigate the clinical course and characteristics of ANCA-associated vasculitis coexisting with cholesterol emboli, we reviewed previous case reports on the development of cholesterol emboli after angioplasty/PCI.

Table 1 summarizes key patient characteristics and outcomes from various studies on CES. Purpura secondary to cholesterol emboli is usually seen on the lower extremities of patients with atherosclerotic vascular disease. They often follow anticoagulant therapy or an invasive vascular procedure, such as an arteriogram, but also occur spontaneously from the disintegration of atheromatous plaques. Associated findings include livedo reticularis, gangrene, cyanosis, and ischemic ulcerations. Multiple-step sections of the biopsy specimen may be necessary to demonstrate the cholesterol clefts within the vessels. The imaging findings and laboratory results corroborate the diagnosis and highlight the potential for significant end-organ damage following vascular interventions in patients with atherosclerotic disease. Continuous monitoring and a multidisciplinary approach will be critical in managing this patient’s condition and preventing further complications.

**Table 1:** Summary of case reports on cholesterol embolization syndrome (CES) M, Male; F, Female; RPGN, Rapidly Progressive Glomerulonephritis; ESRD, End-Stage Renal Disease; HF, Heart Failure

Case Report	Age of the Patient (Years)	Sex	Associated Comorbidities	Smoking Status	ESRD	Death
Tokatli et al. [9]	71	M	Hypertension, Diabetes Mellitus, RPGN	No	No	No
Otsubo et al. [10]	55	M	Hypertension, Hyperlipidemia, Diabetes Mellitus	No	No	No
Nhiri and Bazid [11]	84	M	None	Yes; 62 pack/year	Yes	Yes
Hendrickx et al. [12]	65	F	Severe unstable angina, abdominal aortic aneurysm, and diabetes mellitus	No	No	Yes; 5 months later to HF
Akpala et al. [13]	74	M	Severe coronary artery disease, chronic kidney disease, peripheral vascular disease, carotid artery occlusion, and hyperlipidemia	No	No	No

Set guidelines for treating CES are not available; therefore, the current treatment is primarily supportive care tailored toward the affected organ [13]. Maintenance of hemodynamic stability, pain control, and wound care is crucial in the acute phase. Hemodialysis may be needed for renal failure. Statins are usually considered because of their anti-inflammatory, lipid-lowering, and plaque-stabilizing properties, but the use of other treatments, such as antiplatelets, anticoagulants, and corticosteroids in CES, is controversial [13]. Anticoagulants such as warfarin are usually avoided, as there is concern for increased risk of CES unless there is an underlying condition that requires their use [13]. Currently suggested therapies include fibrinolytic drugs that could potentially prevent crystal clot formation and acute kidney injury without any changes in the crystal component, as well as anti-P2Y12-based antiplatelet therapies that can regulate thrombus stability by preventing platelet aggregation and activation [14]. There is also DNase I anti-thrombotic therapy, an enzyme that prevents thrombus growth by inhibiting fibrin production and adenosine triphosphate (ATP) release from activated platelets [14]. Our patient responded well to the treatment given and continues to follow up.

In conclusion, while biopsy remains the gold standard for confirming CES, the absence of cholesterol clefts in our case represents a significant diagnostic limitation. Nevertheless, the combination of characteristic clinical features, imaging evidence of proximal arterial occlusion, and systemic inflammatory findings provided strong supportive evidence for the diagnosis.

## Conclusions

CES is a rare but serious complication of angioplasty in patients with atherosclerotic disease. We report a 67-year-old male who developed blue toe syndrome and progressive ischemic changes post-PCI, with diagnosis supported by clinical features, imaging, and skin biopsy. Despite supportive care, his condition worsened, emphasizing the importance of early recognition and multidisciplinary management. This case highlights gaps in evidence-based treatment for CES and the need for further research to guide prevention and therapeutic strategies.
